# An Unusual Case of Secondary Hemophagocytic Lymphohistiocytosis in a Patient Diagnosed With Triple Infection: COVID-19, HIV, and Histoplasmosis

**DOI:** 10.7759/cureus.26428

**Published:** 2022-06-29

**Authors:** Masara Touza, Monica Mutyala, Sudipa Chowdhury, Jihad Slim

**Affiliations:** 1 Medicine, Saint George's University School of Medicine, West Indies, GRD; 2 Infectious Diseases, Saint Michael's Medical Center, Newark, USA; 3 Infectious Diseases, Saint Michael's Medical Center/New York Medical College, Newark, USA; 4 Internal Medicine, Saint Michael's Medical Center, Newark, USA

**Keywords:** cytokine storm, antiretroviral therapy, hiv, histoplasmosis, shlh, covid-19

## Abstract

In this study, we present a unique instance of a patient who developed hemophagocytic lymphohistiocytosis secondary to a triple infection with coronavirus disease 2019 (COVID-19), HIV, and histoplasmosis. We emphasize the proinflammatory dysregulations driving the severity of COVID-19 infection in this setting and highlight the importance of early diagnosis and targeted therapy of underlying conditions as a method to increase the chance of survival.

## Introduction

Hemophagocytic lymphohistiocytosis syndrome (HLH) is a pathological entity characterized by T cell-driven cytokine storm secondary to upregulated inflammatory processes that increase cytokine production and hemophagocytosis, which is potentially fatal. HLH may be a primary familial syndrome (pHLH) due to a genetic immune defect or a secondarily acquired syndrome (sHLH) resulting from malignancies, autoimmune diseases, or infections including coronavirus disease 2019 (COVID-19), HIV, and histoplasmosis [1].

The distinctiveness of this case report is that we are presenting a patient with a triple co-infection with COVID-19, HIV, and histoplasmosis leading to sHLH. We have not identified published reports of such a serious blend of pathogens co-occurring in a single setting.

The HLH-2004 diagnostic criteria require patients to either fulfill a molecular diagnosis consistent with HLH or to meet at least five out of eight criteria that comprise the following - (1) fever; (2) splenomegaly; (3) cytopenia affecting at least two lineages; (4) hypertriglyceridemia and/or hypofibrinogenemia; (5) biopsy of bone marrow, spleen, or lymph node demonstrating hemophagocytosis; (6) low or no natural killer (NK) cells; (7) ferritin of at least 500 μg/L, and (8) elevations of soluble cluster of differentiation (CD)25 above age-adjusted, laboratory-specific normal levels (defined as >2 standard deviations from the mean) or at least 2400 U/mL while taking into consideration that hyperferritinemia of >10,000 μg/L increases the probability of HLH diagnosis with sensitivity of 90% and specificity of 96% [1,2]. Even though HIV may as well trigger HLH, opportunistic infections in patients with HIV are the main precipitant for HLH onset.

Based on statistics from WHO data, Honduras accounts for 60% of all AIDS cases in Central America; Honduras makes 17% of its total population [3]. It has also been the second leading cause of hospitalization and death in Honduras [3]. Histoplasmosis was reported to be the most common AIDS-defining illness present in Latin America with an incidence of 0.15 per 100,000 person-years [4].

## Case presentation

The patient is a 26-year-old Honduran male with no past medical history who presented to the emergency department (ED) with multiple symptoms for the past two to three months that include night sweats, fever, fatigue, generalized weakness, malaise, productive cough of clear sputum, exertional dyspnea, nonintentional 20-30 pound weight loss as well as occasional vague abdominal pain. The patient denied chest pain, headaches, hematemesis, hematochezia, melena, or dysuria. He admits to being in a sexual relationship with two female partners.

The patient appeared emaciated, with palpable lymphadenopathy on the right anterior cervical chain, right inguinal area, and possibly small left supraclavicular lymphadenopathy. He was admitted and placed on telemetry for three days, where he spiked fevers >101°F and tachycardic at 140 bpm without ischemic changes on EKG. Initial laboratory tests found the patient positive on PCR for COVID-19 infection. Lab results were also notable for the values listed in Table 1.

**Table 1:** Initial laboratory results of the patient.

Lab finding	Value	Range
Hemoglobin (Hb)	7.9 g/dL	13.5-17.5
Red blood cell count	2.9/uL	4.32-5.72 × 10^6^
Hematocrit	24.4%	38.8-50
Mean corpuscular volume	84.3 fL	81.2-95.1
Mean corpuscular hemoglobin	27.2 pg	27.5-33.2
Mean corpuscular hemoglobin concentration	32.2 g/dL	33.4-35.5
Red cell distribution width	16.9%	11.8-15.6
Bands	16%	0-5
Metamyelocyte 1	2	0
White blood cell count	7.1/uL	4.4-11 × 10^3^
Platelets	186/uL	150-450 × 10^3^
Mean platelet volume	8.4 fL	7.4-11
Nucleated red blood cells	0.04	0-1
Neutrophils	94.6%	40-60
Lymphocytes	2.7%	20-40
Monocytes	2.5%	4-8
Eosinophils	0.1%	1-3
Basophils	0.1%	0-1
Lymphocytes	0.2/uL	1.2-3.4
Absolute neutrophils	6.7/uL	1.4-6.5
Absolute monocytes	0.2/uL	0.11-0.59
Absolute eosinophils	0/uL	0-0.5
Absolute basophils	0/uL	0-0.2
Procalcitonin	0.77 ng/mL	<0.1
Sodium	128 mmol/L	135-145
Aspartate aminotransferase	133 U/L	5-40
Alkaline phosphatase	221 U/L	44-147
Lactate dehydrogenase	726 U/L	122-222
C-reactive protein	10.5 mg/dL	0-0.8
D-dimer	16418 ng/mL	<500
Ferritin	11510 ng/mL	20-250

**Figure 1:**
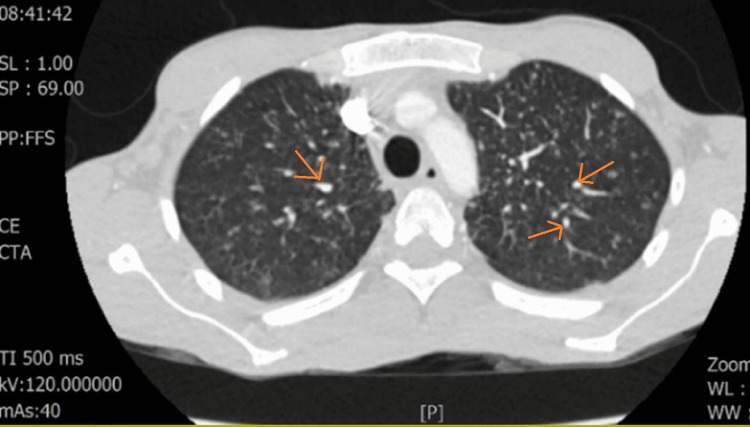
Lung window view demonstrating the bilateral pulmonary nodules indicated with arrows on the patient's chest CT.

Chest x-ray was unremarkable for any acute cardiopulmonary disease. CT of the chest, abdomen and pelvis with oral and IV contrast showed mesenteric and retroperitoneal adenopathy with multiple small ground-glass nodules throughout the lungs, suggestive of a lymphoproliferative disorder such as lymphoma, military tuberculosis (TB), or disseminated histoplasma but atypical for COVID-19 pneumonia (Figure 1).

Initial oxygen (O_2_) saturation was 98% on room air; he did not receive remdesivir nor decadron but was given convalescent plasma and therapeutic heparin anticoagulation. The patient was put in an isolation room while a further investigation took place. Within three days, he desaturated to 89% on room air and was transferred to the intensive care unit to be given remdesivir and decadron. While CT angiogram showed no evidence of pulmonary embolus, it was positive for bilateral hilar and subcarinal lymphadenopathy and mild axillary lymphadenopathy, in addition to findings suggestive of interstitial lung disease. Fibrinogen level was 230 mg/dL, the lower side of the normal range.

The patient refused a diagnostic bone marrow biopsy. The QuantiFERON Gold was negative as well as sputum acid-fast bacillus (AFB). His cluster of differentiation (CD)4 count could not be reported because the absolute lymphocyte count was too low to accurately determine the differentiation of the T cell and B cell subset for the first two test trials. He was found to be HIV-1 positive with a viral load of 511,000. The urine antigen for histoplasmosis and the serum Fungitell assay were positive. Antiretroviral therapy (ART), as well as amphotericin B, was initiated. He was found to have positive urine nucleic acid amplification test (NAAT) for *Chlamydia trachomatis* and was given doxycycline.

Serum Cryptococcus Ag, urine legionnaire’s Ag, and rapid plasma regain (RPR) were negative. His hemoglobin (Hb) and platelet levels dropped, heparin-induced thrombocytopenia antibody (HIT Ab) was negative, and the ferritin trended to a maximum of 93,488 ng/mL. CD25 came elevated at 2129 U/mL (223-710). Triglycerides 220 mg/dL (0-150). The picture for HLH was becoming more noticeable, but soon after adequate treatment, inflammatory markers were noted to be trending down, and the fever was also resolving. Flow cytometry was negative for lymphoma, while CD163 could not be performed on peripheral blood.

Finally, after 19 days of inpatient care, the patient was discharged on ART, fluconazole for histoplasmosis therapy continuation, dexamethasone taper, apixaban for DVT prophylaxis, and Bactrim for pneumocystis jirovecii pneumonia (PJP) prophylaxis.

Nineteen days after discharge, the patient was asymptomatic with a viral load reduced to 500 copies/mL and a T lymphocyte panel showing absolute CD4 helper of 56/uL (359-1519), CD4 of 11.2% (30.8-58.5%) and CD4/CD8 ratio of 0.15 (0.92-3.72).

## Discussion

Even though the bone marrow biopsy could not be carried out for this patient, the criteria for HLH have been met by 5/8. This is likely secondary to the active inflammatory processes occurring because of the COVID-19-driven cytokine storm, superimposed by histoplasmosis infection in the setting of immunosuppression secondary to HIV.

HLH in HIV patients is well-documented, especially with concomitant infections [5]. In adults, HLH-94 protocol treatment, which includes dexamethasone, cyclosporine A, intrathecal therapy, and etoposide, is not as religiously followed as a pediatric HLH case [1]. The protocol is tailored based on the underlying condition causing sHLH [1]. In a patient with HIV, it is recommended to treat the overt inflammation of HLH with corticosteroids with or without IVIG [1]. It is proposed that HIV patients diagnosed with HLH have a poor prognosis [6]. Most of these patients were in an advanced stage of their HIV infection. They were found to have concurrent infections or malignant diseases, foreshadowing a poor recovery rate and prognosis, especially at low CD4 counts [6].

There are documented cases of HLH secondary to histoplasmosis where the mortality rate reported in one of the case series was 38% [7]. Further, 30.7% of the patients were either not diagnosed in the premortem period or were not initiated on a specific treatment regimen [7]. This indicates the value of presenting more cases of HLH associated with histoplasmosis. This underdiagnosed entity of sHLH should be more commonly recognized at the right time and setting to initiate therapy that targets the pro-inflammatory mechanism adequately.

SHLH in patients co-infected with HIV and histoplasmosis has also been well documented. A recent literature review of the case series, published in the Canadian Journal of Infectious Diseases and Medical Microbiology, looked at 65 reported cases of histoplasmosis associated with HLH [8]. They found that 62% of these cases had an underlying HIV infection, with a median CD4 count of 17 cells/μL [8].

There are few publications that discuss HLH in the presence of COVID-19 infection. The symptoms of COVID-19 disease may overlap with those of sHLH, including high fever, cough, dyspnea, myalgia, and fatigue [9]. Laboratory findings usually present in patients with sHLH, such as cytopenia, elevated d-dimer, lactate dehydrogenase (LDH), liver function tests, acute phase reactants, and ferritin, are also commonly found in patients with COVID-19 [9]. However, these lab abnormalities are more prominent in severe COVID-19 patients than in less severe [9]. In non-surviving COVID-19 patients, the d-dimer, serum ferritin, and IL-6 were elevated throughout their clinical course [9]. The cytokine profile resembling sHLH is associated with the severity of COVID-19 [9]. In hospitalized patients with COVID-19, the baseline and maximum numbers of d-dimer activated partial thromboplastin time and prothrombin time were significantly higher in non-survivors compared to survivors [9]. Non-survivors also had higher fibrinogen concentrations at baseline but lower minimum values than survivors [9]. These differences indicate the emergence of organ failure and call for the increased risk of death in patients with COVID-19 [9]. In essence, this image of consumptive coagulopathy due to the proinflammatory cytokine release driving the activation of alternative fibrinolytic pathways in macrophages (via increased release of tissue plasminogen activator), an additional liver failure would exacerbate the coagulopathy present in a patient with a COVID-19 infection, demonstrating a more severe image of sHLH lab findings and thus a higher risk of death from COVID-19 disease [9]. The elevated ferritin and IL-6 indicate that mortality is likely secondary to a virally driven hyperinflammatory pathway [9]. Even though corticosteroids are not routinely recommended in infectious settings since they could exacerbate the presentation, they do prove to be beneficial immunosuppressors in the setting of infection-associated hyperinflammation [9].

After this discussion showing the high risk of death in a patient with either sHLH with HIV, sHLH with histoplasmosis, or sHLH with COVID-19, one would believe that a patient would hardly survive if he presents with a triple infection of COVID-19, HIV, and histoplasmosis causing sHLH. However, our patient, who had severely elevated lab findings, was managed with targeted therapy for his underlying conditions and was able to survive while continuing treatment after being discharged. This again calls for the importance of early diagnosis of underlying conditions and early therapy initiation, especially in the case of sHLH, since early control of the hyperactive immune system could improve the patient’s mortality - even if there is no definite treatment for COVID-19 infection yet.

To identify the group of patients who would benefit and have improved mortality from immunosuppressive therapy, patients with severe COVID-19 may benefit from early screening for hyper-inflammation using the H-score and trending laboratory inflammatory markers [9]. H-score may estimate an individual's risk of having reactive hemophagocytic syndrome as it aids in the detection of hyperinflammatory states in patients with sHLH. This was an interesting opportunity for us to use the H-score in this type of patient. For the patient presented in this case, his H-score totals 234 points (where the cutoff is 169), correlates with a 98-99% probability of a hemophagocytic syndrome, and he would likely benefit from the immunosuppressive treatment (dexamethasone in this case). In other words, in this patient's case, the H-score correlated with the deteriorating inflammatory state. In fact, based on our patient’s T lymphocyte panel and inflammatory markers two weeks after discharge, the dexamethasone, along with the other treatments, did help in improving his deteriorating inflammatory crisis. This also calls for future research opportunities that should further assess the use of this assessment tool, as it may seem to be helpful in certain circumstances.

ART treatment has been playing a role in improving the prognosis of HLH in HIV patients [1,10]. In addition, HLH is solidly fatal without treatment, yet the utilization of immunosuppressive medications in the setting of underlying opportunistic infection can also have fatal outcomes. This poses a challenge in the decision-making process for treating such cases, especially when the patient has an active COVID-19 infection, where dexamethasone therapy has been playing an essential role in treating those patients.

The optimal timing for ART initiation in patients with concurrent HIV/AIDS and histoplasmosis has been an interesting dilemma. Cellular immunity is a key to defense against opportunistic infections; in fact, antiretroviral therapy promotes the immune system's renovation. A study that compared histoplasmosis treatment outcomes in individuals co-infected with AIDS/HIV showed that the response rate was 100% among patients receiving ART, compared with 47% among those who were not receiving ART; they also showed that the cohort given dual antifungal and ART, responded as well as the non-HIV histoplasmosis cohort who were only treated with antifungals [11]. Undiagnosed histoplasmosis in HIV patients may be unmasked with ART initiation through the development of immune reconstitution inflammatory syndrome (IRIS) [12]. For that reason, based on the guidelines published by the Infectious Disease Society of America (IDSA), it is recommended that ART should not be withheld for the concern of developing IRIS in the setting of histoplasmosis treatment, especially that IRIS in the setting of histoplasmosis treatment is considered non-severe and has a rare occurrence [12,13].

In the case of our patient, since the CD4 lymphocyte count was too low even to be determined, we decided to initiate the ART treatment along with amphotericin B and steroids simultaneously. Based on the follow-up of inflammatory markers for our patient, we noticed their decrease within the first week of treatment initiation and elevated CD4 counts 19 days after discharge. However, we should remember that using dexamethasone in this patient has likely been a factor that prevented the development of IRIS.

Finally, it is interesting to witness the rebuilding of the patient’s immune system after putting together an appropriate therapy to treat his multiple infections. After having an extremely low absolute lymphocyte count on two consecutive tests during hospitalization, the T lymphocyte panel began showing higher numbers of cells two weeks later.

## Conclusions

sHLH caused by COVID-19 is an underrecognized form of the cytokine storm. It would be essential to emphasize immunologic dysregulations driving the proinflammatory presentations, especially in patients with COVID-19, because a helpful approach in managing this type of patient is creating a therapeutic approach that targets these proinflammatory drivers. In conclusion, because prompt initiation of treatment of AIDS-defining illnesses may improve outcomes, it is crucial to choose adequate treatment options while considering drug-drug interactions. In the setting of HLH, treatment does not solely include focusing on the underlying triggers. It is important to support the treatment of HLH by giving steroids. It is recommended to start antiretroviral therapy as early as possible to support the body’s cellular immune and not pause it to give antifungal treatment for the concern of developing inflammatory-driven IRIS.
